# Relationship of lymphovascular invasion with lymph node metastasis and prognosis in superficial esophageal carcinoma: systematic review and meta-analysis

**DOI:** 10.1186/s12885-020-6656-3

**Published:** 2020-03-04

**Authors:** Jinxin Yang, Zhouyi Lu, Lintao Li, Yong Li, Yulong Tan, Dekang Zhang, An Wang

**Affiliations:** 1 Department of Radiation Oncology, Sichuan Cancer Hospital and Institute, Sichuan Cancer Center, School of Medicine, University of Electronic Science and Technology of China, Chengdu, Sichuan China; 2 Department of Thoracic Surgery, Huashan Hospital, Fudan University, Shanghai, China

**Keywords:** Lymphovascular invasion, Lymph node metastasis, Prognosis, Superficial esophageal carcinoma

## Abstract

**Background:**

The development of tumor cells inside the lymphatics or blood vessels is known as lymphovascular invasion (LVI). The correlation between LVI, lymph node metastasis (LNM), and the diagnosis of superficial esophageal carcinoma (SEC) remains unclear.

**Methods:**

We searched Embase, PubMed, Web of Science, and Cochrane Library databases for prospective articles to better understand the relationship between LVI, LNM, and SEC diagnosis.

**Results:**

We included 23 articles containing data for 4749 patients (range: 54–598) in our meta-analysis. The hazard ratio between LVI and overall survival (OS) was 1.85 with 95% confidence interval (CI) (1.10–3.11, *P* = 0.02). LNM rate was higher in SEC patients with LVI than SEC patients without LVI (univariate: OR = 4.94, 95% CI: 3.74–6.53, *P* < 0.0001; multivariate: OR = 5.72, 95%CI: 4.38–7.4, *P* < 0.0001). No obvious publication was found.

**Conclusions:**

The results indicate that LVI plays a dominant role in the prognosis of LNM in SEC and in the prognostic prediction for SEC.

## Background

Superficial esophageal carcinoma (SEC) can be classified as submucosal (T1b), mucosal (T1a), or intraepithelial (Tis) irrespective of lymph node metastasis (LNM). Patients suffering from SEC have a better chance of survival after esophagectomy compared to those with advanced esophageal carcinoma (EC). According to the Japanese criteria, the depth of tumor invasion is subclassified into six layers. The mucosa is subdivided into the intraepithelial (m1) region, lamina propria (m2), and muscularis mucosa (m3) while the submucosa is homogeneously classified into three sections: inner (sm1), middle (sm2), and deep submucosa (sm3) [1]. The prognostic factors for EC include the histology type, tumor size, grade category, invasion depth, blood vessel and lymphatic vessel permeation, as well as LNM and distant metastasis [2]. EC patients with LNM frequently have an adverse prognosis. Therefore, the impact of LVI on LNM and prognosis requires attention.

The development of tumor cells inside the lymphatics or blood vessels is known as lymphovascular invasion (LVI). Lymphatic vessels are believed to play a crucial role in LNM and their presence increases the micro-metastatic risk in locoregional malignancy [3]. Though lymph node metastasis via LVI or lymphatic vessels has not been confirmed [4], lymphatic vessels are known to provide entry for the penetration of tumor cells [5]. Some studies have provided evidence of an association between LVI and LNM in SEC. Nonetheless, the impact of LVI on OS and LNM in SEC requires investigation. Thus, we conducted a meta-analysis to obtain additional insight into the correlation between LVI, LNM, and prognosis in SEC.

## Methods

### Search strategy

We searched the Embase, PubMed, Web of Science, and Cochrane Library databases for prospective articles. The search terms used were (lymphovascular invasion (LVI) OR lymph vessel invasion OR angiolymphatic invasion OR lymphatic invasion) AND (superficial esophageal cancer (SEC) OR submucosal esophageal carcinoma OR mucosal esophageal cancer OR T1 esophageal carcinoma). We conducted a manual search of the results to identify the prospective studies relevant to our investigation. We then performed preliminary screening by checking the titles followed by the abstracts. Relevant studies were confirmed after reviewing the full text. In the present study, we regarded lymphatic invasion as LVI.

### Exclusion and inclusion criteria

Studies were considered eligible based on the following criteria: (1) SEC; (2) hazard ratio (HR) for prognosis and odds ratio (OR) for LNM; (3) papers published in English; (4) the latest or most relevant articles published by the same group/author.

The exclusion criteria were as follows: (1) duplicate conference papers, reviews, reports, abstracts, and letters; (2) studies about other cancer types, animal models, esophageal cancer cell lines, and treatment methods; (3) lack of data on prognosis or LNM; (4) studies published in languages other than English; (5) esophagogastric junction cancer (EJC).

### Preliminary review of studies and quality assessment

Each selected article was reviewed by two independent authors based on the exclusion and inclusion criteria above. When a discrepancy arose, a third author was involved to resolve the differences. Quality assessment was performed using the Newcastle-Ottawa Scale (NOS) [6] and all articles included scored a minimum of five points on the NOS. Researches about prognosis were assessed by critical appraisal of prognostic studies (https://www.cebm.net/wp-content/uploads/2018/11/Prognosis.pdf). The detailed quality assessment of these studies was displayed in a Table 1.

**Table 1:** The detailed quality assessment of prognostic studies

Author	Years Included	Region	Comment 1	Comment 2	Comment 3	Comment 4	What are the results
Leggett (2015) [7]	1995-2011	USA	Yes	Yes	Yes	Yes	Survival curve, CI is narrow, conclusion is promotable
Yamashina (2013) [8]	1995-2010	Japan	Yes	Yes	Yes	Yes	CI is relative marrow, conclusion is promotable
Tanaka (2014) [9]	1988-2010	Japan	Yes	Yes	Yes	Yes	CI is narrow, conclusion is promotable
Xue (2018) [10]	1990-2004	China	Yes	Yes	Yes	Yes	CI is relative marrow, conclusion is relative promotable

*CI* Confidence interval

### Data extraction

Two independent authors collected data from the studies. The following information was extracted: surname of the first author, follow-up years, region, sample size for the research, treatment characteristics, histology type, depth of invasion, staining methods, the percentage of patients with LVI, information about OS, and LNM and NOS scores. All of the collected information is listed in Table 2. Discrepancies among authors were resolved.

**Table 2:** Characteristics of studies included in out meta-analysis

Author	Years Included	Region	No.	Treatment Characteristic	Pathology	Depth of Invasion	Staining	Indicator (No.)	Including Statistics	NOS Scores
Jia (2016) [11]	2010-2015	China	93	Esophagectomy and lymphadenectomy	SCC/Others	M1-SM3	NM	LVI(28)	LNM	5
Sepesi (2010) [12]	2000-2008	USA	54	Esophagectomy and lymphadenectomy	AD	SM	NM	LVI(7)	LNM	5
Leggett (2015) [7]	1995-2011	USA	269	EMR followed by ablative techniques	AD	LP-SM	H&E	LVI(53)	OS	6
Huh (2017) [13]	1996-2015	Korea	275	187 Esophagectomy and 88 ER (Esophagectomy or ER)	SCC	M-SM	H&E	LVI(36)	LNM	6
Zhou (2016) [14]	2008-2015	China	498	Esophagectomy with lymphadenectomy	SCC	M1-SM3	H&E/IHC	LI(16/412)	LNM	7
Moon (2014) [15]	2009-2012	Korea	104	Esophagectomy with lymphadenectomy	SCC	M1-SM3	H&E	LVI(13)	LNM	6
Mitobe (2013) [16]	1990-2009	Japan	110	106 Esophagectomy with lymphadenectomy, 4 esophagectomy follwed ER and lymphadenectomy	SCC	LP-SM3	IHC	LI(42)	LNM	6
Nentwich (2014) [17]	1994-2009	Germany	67	Esophagectomy	SCC/AD	SM	NM	LI(16/61)	LNM	5
Raja (2011) [18]	1983-2010	USA	120	Esophagectomy	SCC/AD	SM	NM	LVI(26)	LNM/OS	5
Nakajima (2002) [19]	1985-1995	Japan	84	Esophagectomy with lymphadenectomy	SCC	SM	IHC	LI(60)	LNM	6
Choi (2011) [20]	1991-2009	Korea	190	Esophagectomy with lymphadenectomy	SCC	M1-SM3	H&E	LVI(39)	LNM	7
Tajima (2000) [21]	1968-1996	Japan	240	Esophagectomy with lymphadenectomy	SCC	LP-SM	H&E	LI(39/186)	LNM	6
Chiba (2010) [22]	1992-2008	Japan	110	107 underwent esophagectomy, 3 patients underwent ER followed esophagectomy	SCC	M-SM	IHC	LI(46)	LNM	6
Yamashina (2013) [8]	1995-2010	Japan	402	EMR or ESD, some patients received surgery after ER	SCC	EP-SM2	NM	LVI(33)	OS	5
Xue (2012) [23]	1990-2004	China	271	Esophagectomy	SCC	M2-SM3	IHC	LI(51)	LNM	7
Ancona (2008) [24]	1980-2006	Italy	98	Esophagectomy with lymphadenectomy	SCC/AD	M1-SM3	NM	LI(34)	LNM	5
Li (2013) [25]	2006-2011	China	189	Esophagectomy with lymphadenectomy	SCC	M1-SM3	NM	LVI(22)	LNM	5
Qi (2016) [26]	2009-2014	China	258	Esophagectomy with lymphadenectomy	SCC	SM	H&E	LVI(18)	LNM/OS	6
Wang (2016) [27]	2002-2014	Japan	598	Esophagectomy with lymphadenectomy	SCC	M-SM	H&E/IHC	LI(62/228)	LNM	6
Kim (2008) [28]	1994-2006	Korea	200	Esophagectomy with lymphadenectomy	SCC/AD	M-SM	NM	LI(33)	LNM	5
Tanaka (2014) [9]	1988-2010	Japan	145	Esophagectomy with lymphadenectomy	SCC	SM1-SM3	NM	LVI(84)	OS	5
Zhuge (2018) [29]	2006-2016	China	175	Esophagectomy with lymphadenectomy	SCC	SM1-SM3	NM	LVI(32)	LNM	6
Xue (2018) [10]	1990-2004	China	199	Esophagectomy with lymphadenectomy	SCC	M2-SM3	IHC	LVI(27)	OS	6

*LVI* Lymphovascular Invasion, *LI* Lymphatic invasion

*ER* Endoscopic resection, *EMR* Endoscopic mucosal resection, *ESD* Endoscopic submucosal dissection

*SCC* Squamous cell carcinoma, *AD* Adenocarcinoma, *OS* Overall survival

*EP* Epithelium, *M* Mucosa, *SM* Submucosa, *LP* Lamina propria, *NM* Not mentioned

*H&E* Hematoxylin-eosin, *IHC* Immunohistochemical

### Statistical analysis

We investigated the correlation between LVI, prognosis, and LNM in SEC patients. HR and OR were effective for the prognosis and LNM with 95% CI individually. Worse prognosis for SEC was indicated by an HR value > 1. Cochrane’s Q test (Chi-squared test; Chi2) and the I^2^ metric were used to test the heterogeneity of the pooled results. I^2^ < 25% indicated no heterogeneity; I^2^ = 25–50%, moderate heterogeneity; I^2^ = 50–75%, medium heterogeneity; and I^2^ > 75%, extreme heterogeneity. We used a fixed-effect model (the Mantele Haenszel method) for I^2^ < 50% with *P* > 0.05 in this meta-analysis. If not, a random-effect model was appropriate for our analysis. We used meta regression and subgroup analysis to explore heterogeneity when necessary [18]. Begg’s test was used to assess publication bias. Two-tailed tests were used to calculate the *P* value and *P* ≤ 0.05 was considered statistically significant. Statistical analysis was performed using the Stata/SE version 12.0 for Windows (Stata Corporation, College Station, TX, USA).

## Results

### Characteristics of studies

**Fig. 1:**
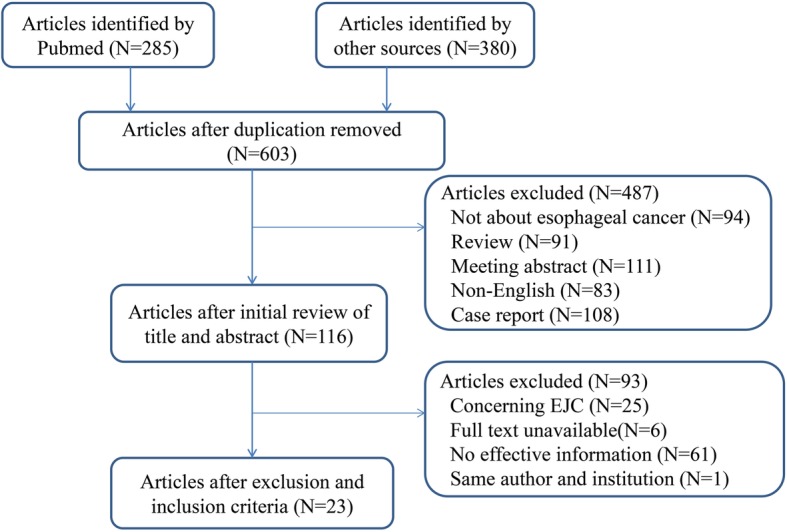
Flow chart showing the literature collection procedure for included studies

We retrieved 603 articles after removing duplicates but excluded 487 articles that were either case reports or only abstracts. A few of the excluded articles were review articles and others contained information about other cancer conditions. Articles published in languages other than English were also excluded. We identified 116 potential articles for full-text review. We excluded 93 articles for the following reasons: 25 were about EJC; 67 lacked data relevant to LVI, prognosis, or LNM; and retrieval of the full text was not possible for six articles; one was excluded due to the same author and institution. The remaining 23 articles, which included information for 4749 patients (range: 54–598), were included in the meta-analysis (Fig. 1). Table 2 shows detailed information about the studies. All studies included in this meta-analysis were rated with a minimum of five stars based on the NOS.

Six studies provided survival information between LVI and prognosis. Two studies reported the association between LVI and prognosis with univariate Cox proportional hazards analysis in included studies [18, 26]. Four of included studies suggested the association between LVI and prognosis was not significant in SEC patients [8, 9, 18, 26]. The rest two studies showed LVI was a poor prognostic indicator in SEC patients [7, 10].

Sixteen studies provided information on LVI from multivariate analysis of LNM cases. Eight studies provided information on LVI from univariate analysis. One study using univariate analysis reported a *p* value of 0.049 [12].

### LVI impact on OS

**Fig. 2:**
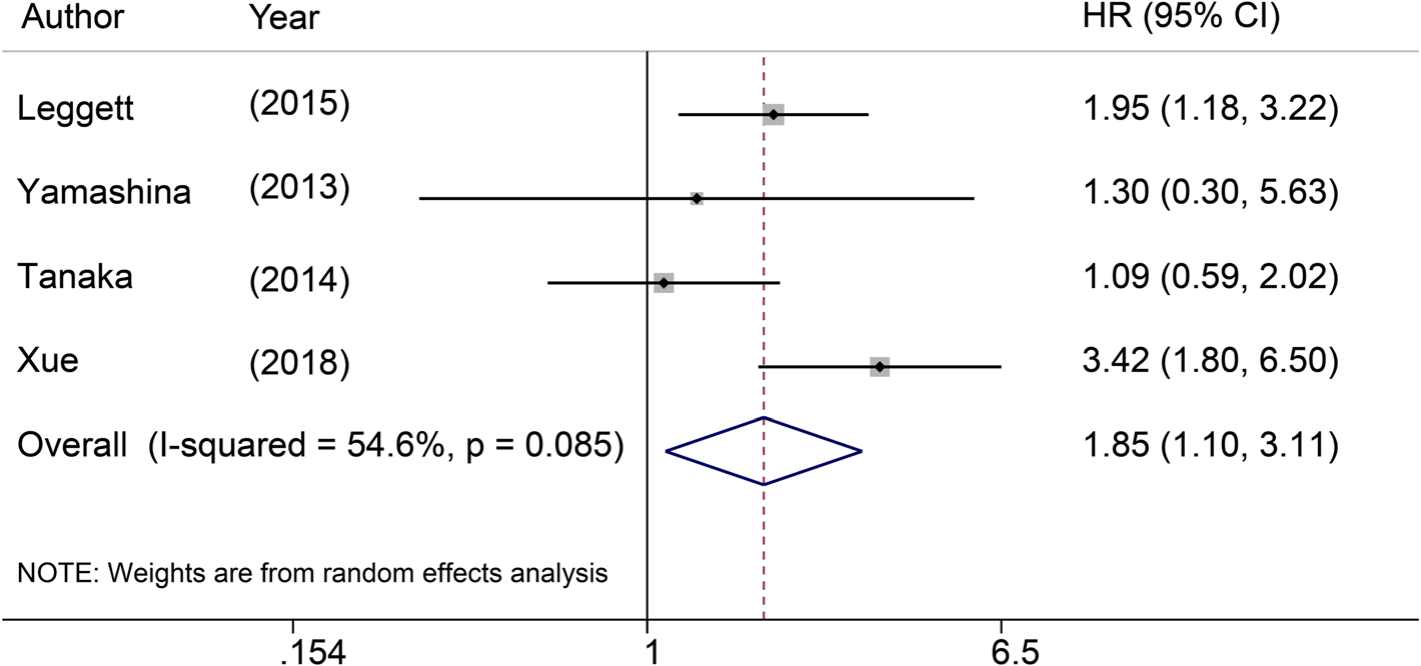
Forrest plot showing pooled HR for OS in patients with LVI

2We included 4 eligible studies containing 1005 patients from multivariate analysis in our meta-analysis. The pooled HR was 1.85 with 95% CI (1.10–3.11, *P* = 0.02) and the pooled OS showed medium heterogeneity based on random effect model (I^2^ = 54.6%, *P* = 0.085, Fig. 2).

### Association between LVI and LNM

**Fig. 3:**
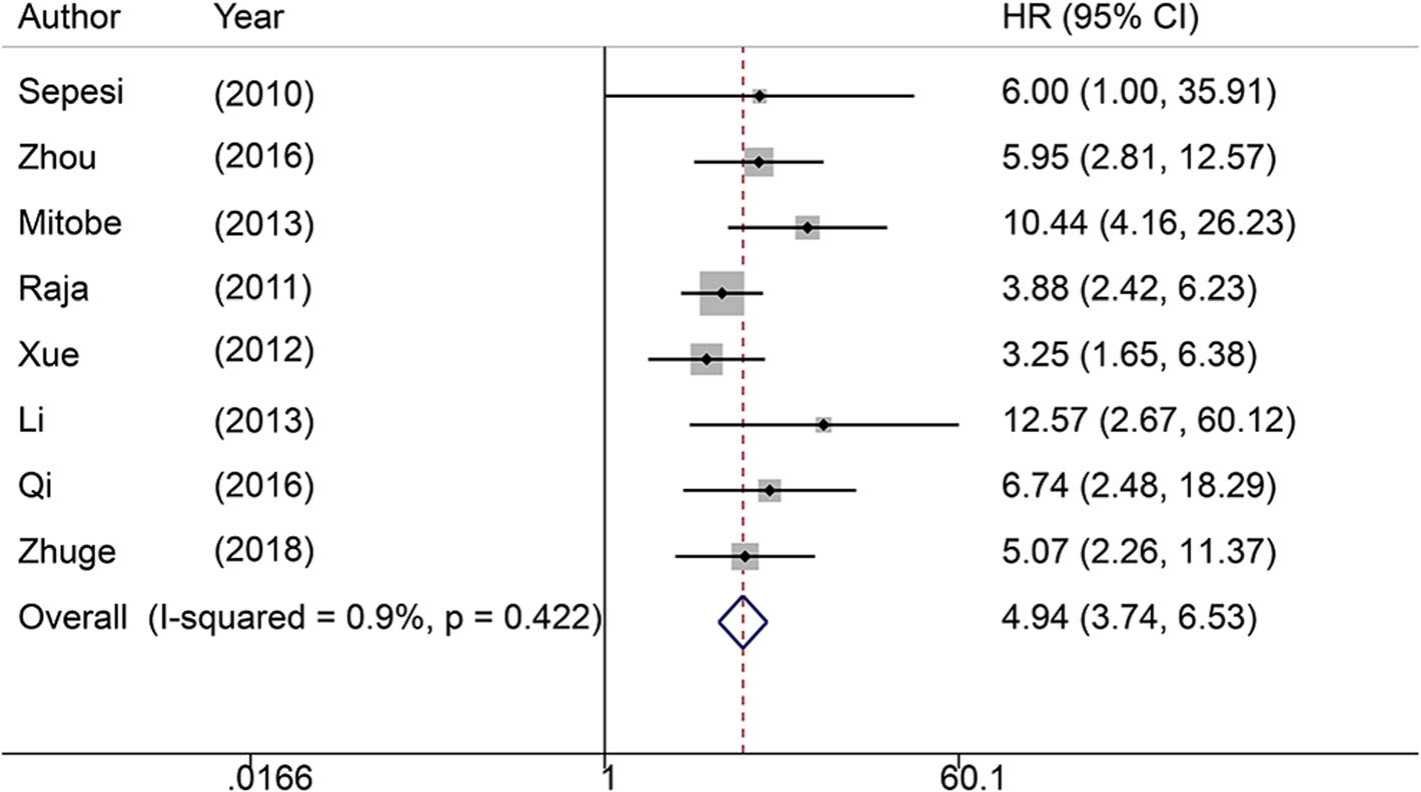
Forrest plot showing pooled OR for LNM in patients with LVI from univariate analysis

**Fig. 4:**
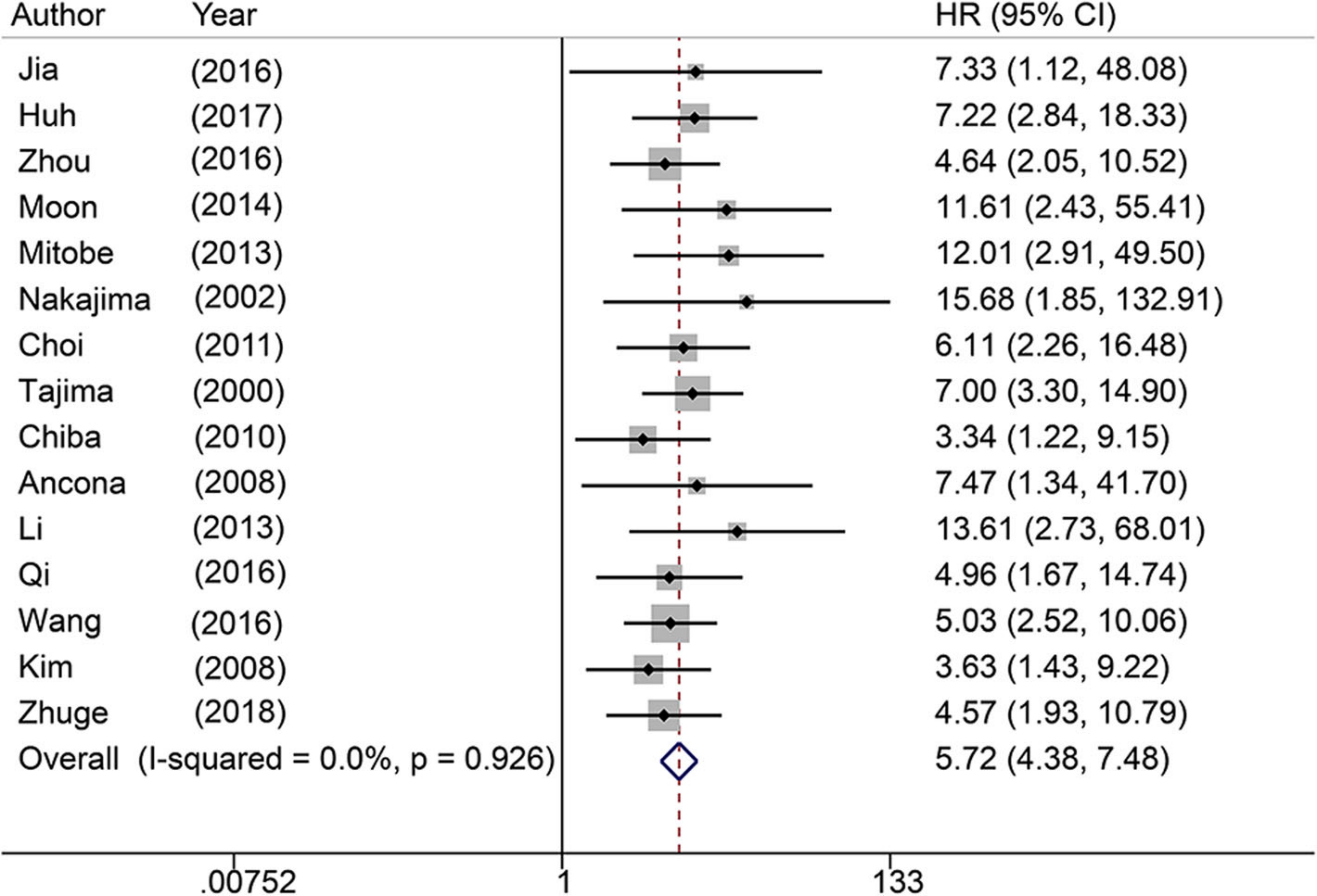
Forrest plot showing pooled OR for LNM in patients with LVI from multivariate analysis

The pooled results showed that patients in the LNM-positive group had an advanced LVI detection rate (OR = 4.94, 95% CI: 3.74–6.53, *P* < 0.0001, Fig. 3) in univariate analysis. The combined results exhibited no heterogeneity (I^2^ = 0.9%, *P* = 0.422). The pooled results from 20 studies in multivariate analysis suggested that LVI significantly increased the risk for LNM (OR = 5.72, 95% CI: 4.38–7.48, *P* < 0.0001, Fig. 4) with no heterogeneity (I^2^ = 0%, *P* = 0.926).

### Publication bias of included studiessl

**Fig. 5:**
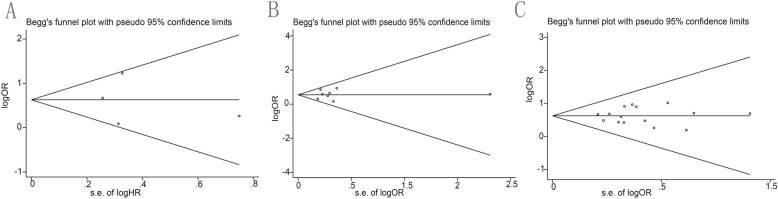
The funnel plots of publication bias, **a** OS publication bias; **b** Bias of LNM on univariate; **c** Bias of LNM on multivariate

There was no evidence of publication bias for OS as demonstrated by Begg’s test (*P* = 1) or for LNM (multivariate: *P* = 0.961; univariate: *P* = 0.805). The funnel plots were displayed in Fig. 5.

## Discussion

Our study demonstrated that SEC patients with LVI have a poor OS (HR = 1.85, 95% CI: 1.10–3.11, *P* = 0.02; I^2^ = 54.6%, *P* = 0.085). LVI significantly reduces OS in patients with SEC. This conclusion should be clarified with caution due to medium heterogeneity. Additionally, LVI and LNM are strongly correlated (univariate: OR = 4.94, 95% CI: 3.74–6.53, *P* < 0.0001, I^2^ = 0.9%, *P* = 0.422; multivariate: OR = 5.72, 95% CI: 4.38–7.4, *P* < 0.0001; I^2^ = 0%, *P* = 0.926) in patients suffering from SEC. These results suggest that LVI is an important prognostic factor for patients with SEC with regard to predicting LNM and survival.

SEC is similar to the esophageal tumors, which are limited to the mucosal layer (T1, T0) and include high-grade dysplasia, intramucosal cancer (T1a), and tumors infiltrating the submucosa (T1b) [30]. .Reports state that patients with T0 (0% chance) or T1a (1–2% chance) esophageal cancer have a minimal risk of local LNM [31]. There is no specific standard available for the detection of LVI. However, the identification of tumor cells in the lymphatic vessels, arteries, or veins during pathological evaluation of specimens indicates LVI. The condition is an independent prognostic factor of LNM in malignant tumors causing lung, prostate, breast, and esophageal cancer. However, the role of LVI in SEC has not been clarified to date. Additionally, the impact of LVI in SEC on OS and LNM has not been assessed using meta-analysis in the past. Therefore, we conducted this study by analyzing data for 4854 patients reported in 24 eligible articles retrieved from PubMed and other relevant sources. We demonstrated LVI relevance in LNM and the prognosis for patients with SEC. According to a literature review, our work is the first systematic review and meta-analysis on LVI relevance in LNM and prognosis in patients with SEC.

During the early stage of esophageal cancer, LVI is regarded as a potential prognostic factor in predicting LNM. Current research has demonstrated that patients with T1b esophageal cancers without LVI have a significantly higher survival rate up to 5 years higher those with LVI [32]. A larger cohort study revealed that LVI has a significant effect on the prognosis after resection for ESCC [33]. Our study shows that SEC patients with LVI have a poor OS (HR = 1.62, 95% CI: 1.17–2.26, *P* = 0.004, I^2^ = 0.0%), and LVI significantly increases the risk of LNM in SEC (univariate: OR = 5.26, 95% CI: 4–6.91, *P* < 0.0001, I^2^ = 30.2%; multivariate: OR = 5.7, 95% CI:4.43–7.33, *P* < 0.0001; I^2^ = 16%). Reports describing the relationship between LVI, LNM, and OS in SEC indicate that LVI raises the possibility of LNM, leading to a poor OS.

Esophagectomy and other non-surgical options including chemotherapy and radiotherapy are the mainstream treatments for esophageal cancer. However, endoscopic resection (ER) is the diagnostic and radical choice for the treatment of SEC with a low possibility of LNM. The Japan Esophageal Society published a guideline in 2014 recommending ER as the best treatment option for T0 and T1a lesions located within the limits of the mucosal layer and not associated with LNM. The treatment can still be applied for lesions that infiltrate the muscularis mucosae or the inner submucosa (T1b-SM1) but the risk of LNM exists for these cases. Hence, other classifications for superficial carcinomas (T1b-SM2 and T1b-SM3) should not be treated with endoscopy alone due to the high rates of metastasis [34]. ER can be classified as endoscopic mucosal resection (EMR) or endoscopic submucosal dissection (ESD). All visible neoplasms are removed by EMR for definitive histopathological staging. However, EMR is ineffective compared to ESD in terms of en bloc resection of large lesions. The largest lesion amenable to en bloc resection with the EMR device is approximately 15 mm [35, 36] whereas en bloc resection can be achieved with ESD regardless of the size of neoplastic lesions [36]. Furthermore, several studies have reported that ESD has a higher R0 resection rate and a lower local recurrence rate compared to EMR. Therefore, ESD is considered the standard for ER treatment of ESCC [37–39]. Esophagectomy, the main surgical treatment for EC, was compared with ER treatment and the results revealed that T1b lesions were managed endoscopically with no impact on survival [40–42]. Therefore, ER is preferable to surgery and also appears to be an optimal first-line treatment for early esophageal cancer.

This study does have some limitations. First, we used only studies published in English for our meta-analysis. Consequently, studies reporting negative results may have been overlooked. Next, the stages, treatment, staining method, and adjuvant therapy differed for each study. In addition, the heterogeneity of OS was medium. The subgroup analysis was unable to carry out due to limited studies. Few studies provided Kaplan-Meier curves and we calculated the HR and 95% CI where necessary. Therefore, we strongly recommend interpreting the results with caution.

## Conclusions

SEC patients with positive LVI indicated poor prognosis compared with patients without LVI. Therefore, the association between LVI and LNM in SEC patients was close.

## Data Availability

The data sets used and analyzed during the current study available from the corresponding author on reasonable request.

## Abbreviations

EC: Esophageal carcinoma
EJC: Esophagogastric junction cancer
EMR: Endoscopic mucosal resection
ER: Endoscopic resection
ESCC: Esophageal squamous cell carcinoma
ESD: Endoscopic submucosal dissection
HR: Hazard ratio
LNM: Lymph node metastasis
LVI: Lymphovascular invasion
NOS: Newcastle-Ottawa Scale
OS: Overall survival
SEC: Superficial esophageal carcinoma
